# A Path Loss and Shadowing Model for Multilink Vehicle-to-Vehicle Channels in Urban Intersections

**DOI:** 10.3390/s18124433

**Published:** 2018-12-14

**Authors:** Mikael G. Nilsson, Carl Gustafson, Taimoor Abbas, Fredrik Tufvesson

**Affiliations:** 1 Deptartment of Electrical and Information Technology, Lund University, Box 118, SE-221 00 Lund, Sweden; Fredrik.Tufvesson@eit.lth.se; 2 Volvo Car Corporation, SE-405 31 Göteborg, Sweden; taimoor.abbas@volvocars.com; 3 SAAB AB, SE-582 54 Linköping, Sweden; carl.gustafson@saabgroup.com

**Keywords:** Vehicle-to-Vehicle, channel modeling, path loss, shadow fading, obstruction, large scale fading, multiple links, diversity, correlation

## Abstract

The non line-of-sight (NLOS) scenario in urban intersections is critical in terms of traffic safety—a scenario where Vehicle-to-Vehicle (V2V) communication really can make a difference by enabling communication and detection of vehicles around building corners. A few NLOS V2V channel models exist in the literature but they all have some form of limitation, and therefore further research is need. In this paper, we present an alternative NLOS path loss model based on analysis from measured V2V communication channels at 5.9 GHz between six vehicles in two urban intersections. We analyze the auto-correlation of the large scale fading process and the influence of the path loss model on this. In cases where a proper model for the path loss and the antenna pattern is included, the de-correlation distance for the auto-correlation is as low as 2–4 m, and the cross-correlation for the large scale fading between different links can be neglected. Otherwise, the de-correlation distance has to be much longer and the cross-correlation between the different communication links needs to be considered separately, causing the computational complexity to be unnecessarily large. With these findings, we stress that vehicular ad-hoc network (VANET) simulations should be based on the current geometry, i.e., a proper path loss model should be applied depending on whether the V2V communication is blocked or not by other vehicles or buildings.

## 1. Introduction

The propagation channel characteristics for ad-hoc Vehicle-to-Vehicle (V2V) communication are fundamentally different from typical infrastructure-based cellular communication due to the higher mobility and the differences in propagation environments around the antennas. During the last decade, considerable effort has been made to understand and model V2V channel characteristics [1,2,3,4,5]. One aspect where little attention has been paid is the modeling of multilink cases, i.e., communication between with one or several transmitters and multiple receivers or vice versa. Exceptions to this include [6], where simultaneous analysis of the communication links was performed between four cars to capture the joint behavior of the shadow fading process in multiple links only for highway scenarios.

The urban environment, especially street intersections, is one of the most safety critical scenarios for V2V safety applications. The German automotive organization ADAC recently reported that more than two people die every day in road accidents at urban intersections [7]. There has been extensive research into advanced driver assistance (ADAS) systems where the on-board sensors, e.g., radars, cameras, and lidars, are utilized to detect potential hazards and reduce the number of road accidents and fatalities. The urban scenario is challenging even for such ADAS systems because of non line-of-sight (NLOS) situations where the on-board sensors have limited functionality and cannot detect the objects. This impairment of on-board sensors can be overcome with V2V communication, by contributing additional information to the sensor fusion systems of the vehicles to improve the safety around the corners. Several V2V collision avoidance applications [8,9] and virtual traffic light applications [10,11] have been proposed for this scenario to improve traffic safety and efficiency at street intersections.

A number of research papers have been published to characterize, model, and validate the V2V channel in street intersections of various types and geometries. Among the first few analytical as well as empirical studies to characterize the V2V channel at the street intersections were [12,13,14], where the authors analyzed the basic channel and directional properties. Later on, several analytical and empirical studies were made to further analyze the channels in urban intersections for line-of-sight (LOS) and NLOS situations in terms of spatial and temporal characteristics, distance between the transmitter and receiver, path loss, and fading [15,16,17,18].

A path loss model for the NLOS scenario based on extensive measurements was presented by Mangel et al. [15]. Mangel’s model was validated with an independent set of data in [19], which confirmed that the model is applicable to a wide range of street intersections with some limitations. The model is, however, not generic in the sense that it does not take into account that the vehicles blocked by buildings might also have vehicles in front that further block the multipath components, and the model is non-reciprocal. Another extensive V2V measurement campaig, with channel sounding equipment in an urban environment was presented in [16], but the NLOS case was left for future work. Ref. [17] addressed spatial properties and it was found that the urban intersections due to rich multipaths and special geometry show a high root-mean-square (RMS) delay spread, especially for short distances, as well as a waveguide effect of the propagation along the streets. Related work in [18] concluded that the urban street intersections impose much more challenging and diverse propagation channel conditions for V2V communication than that in highway or rural environments.

For realistic performance evaluation of V2V communication systems, it is crucial that the channel models used for system simulations are realistic and cover relevant details [20,21,22,23]. There are gaps in knowledge, and no model to date covers all important aspects of the NLOS case. Despite all the efforts, the NLOS case in urban intersections is still one of the most unexplored scenarios for V2V channel modeling. As stated in one of the deliverables from the 5G project METIS [24], the ITU-R UMi path loss model does not fit the NLOS case of V2V communication for any of the investigated frequencies. Most of the studies mentioned earlier, with some exceptions, have focused only on a single link, i.e., one transmitter (TX) and one receiver (RX) and often with the same type of vehicle. To the authors’ best knowledge, no study has analyzed the simultaneous impact of multiple links between different types and sizes of vehicles with different antenna configurations in the urban NLOS case, which is the main topic of interest here.

This paper fills the gap in knowledge by providing path loss and fading parameters for vehicles of different kinds and sizes, addressing multilink shadowing effects in urban intersections. In the paper, we present an alternative NLOS path loss model based on V2V channel measurements in two urban intersections between six vehicles. The model is reciprocal and it also take into account whether the communicating vehicles are obstructed by other vehicles or not. Finally, auto-correlation properties of a single link and cross-correlation properties of the large scale fading between different links are analyzed. The correlation distances of the shadowing process and the cross-correlation of the same process between multiple links are analyzed and parametrized to enable realistic network simulations.

The rest of the paper is organized as follows. In Section 2, the methodology is described, covering the measurement description, data processing, and path loss modelling. Section 3 addresses the results, followed by a disucssion in Section 4. Finally, the conclusions in Section 5 wrap up the paper.

## 2. Method

### 2.1. Measurement Description

**Figure 1:**
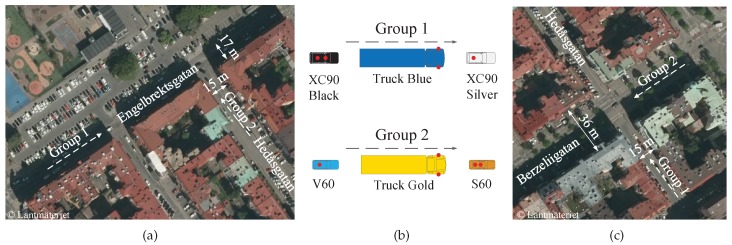
Test scenarios: (**a**) Yngve, a T-junction with buildings only in two corners. (**c**) Xerxes, an X-junction with buildings in all four corners. (**b**) shows the configurations of the two groups where the red dots show the antenna location(s) on each vehicle.

**Figure 2:**
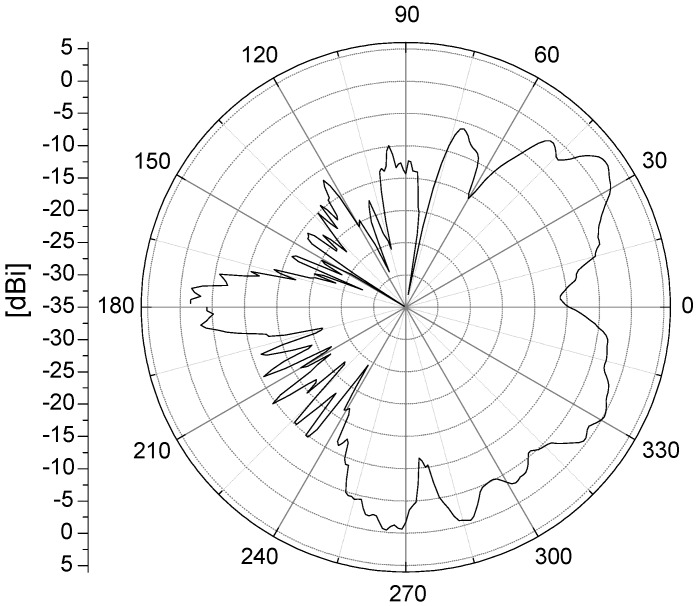
Antenna gain pattern of the XC90 Black Front antenna. A pattern that is represenative when mounting 5.9 GHz antennas on vehicles that are far from omni-directional. The front of the car is in the direction of zero degrees.

**Figure 3:**
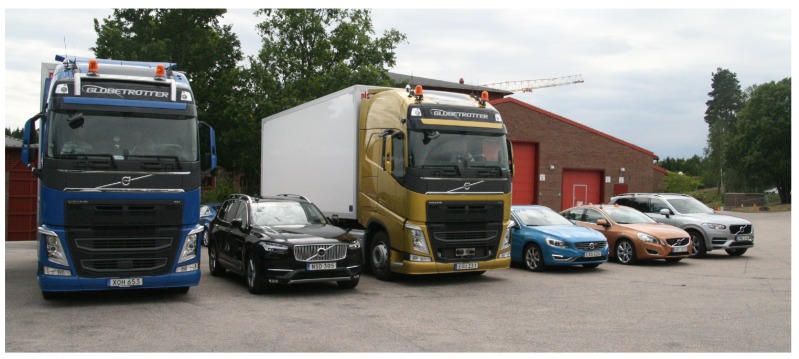
The six vehicles used during the measurements, from the left: Truck Blue, XC90 Black (large SUV), Truck Gold, S60 (mid size sedan), V60 (mid size wagon), and XC90 Silver.

Measurements were performed to capture the joint (simultaneous) behavior of the link gain between six vehicles with one or two antennas on each, in total 41 links, in an urban environment. The six vehicles from AB Volvo and Volvo Cars were divided into two groups driven in the following order: (1) XC90 Silver, Truck Blue, and XC90 Black; and (2) S60, Truck Gold, and V60. The two groups were driven on two orthogonal streets with the purpose of meeting at the intersection called *Yngve*, (lat N 57.70071, long E 11.98203) in Gothenburg, Sweden, or at the intersection called *Xerxes* (lat N 57.69931, long E 11.98404). The measurements took place over two cloudy days in June 2016, and several measurement iterations were performed. The test scenarios and the group configurations together with the antenna location(s) on each vehicle are shown in Figure 1. In Yngve, group 1 drove on Engelbrektsgatan from the left, passing the intersection of Hedåsgatan. Group 2 drove on Hedåsgatan towards the intersection and then stopped. The measurement scenario ended either when group 1 left Engelbrektsgatan or when group 2 turned right into the same street. In Xerxes, group 1 stood still on Hedåsgatan, close to the intersection of Berzeliigatan, or drove very slowly towards the intersection. Group 2 approached Xerxes from the right, and when reaching the intersection, group 2 turned right into Hedåsgatan; after that the measurement scenario ended. The street widths are marked in the figures. The distances from the lane to the facade of the buildings were 7.0 m on Engelbrektsgatan, 7.5 m on Hedåsgatan, and 16 m on Berzeliigatan. All vehicles were equipped with transceivers from a V2V vendor comprising dedicated chipsets from a large manufacturer using the communication standard IEEE 802.11p [25] and antennas designed for 5.9 GHz (see Figure 2). In Figure 3, the six vehicles are shown, while details of the test setup are shown in Table 1. The received signal strength indicator (RSSI), time, GPS coordinates, and videos were recorded from each vehicle. The measurements were conducted with a speed up to 50 km/h (14 m/s), but the speed was mostly lower due to the dense traffic. The test environment was chosen to characterize a rich scattering environment.

### 2.2. Data Processing

All transceivers were synchronized with GPS and they reported RSSI values with a frequency of 10 Hz with a resolution of 0.5 dB when packets were decoded correctly. The log files (RSSI, time and GPS-coordinates) from the transceivers were synchronized manually in time with the videos. By watching the videos, the cases of LOS, obstructed LOS (OLOS), and NLOS communication were identified to support detailed analysis and modeling. Each RSSI value was influenced by the relative distance between TX and RX as well as the small and large scale fading effects. Since the speeds varied greatly during the measurements, we had to made a trade-off between reducing the small scale fading and the effective sampling rate in time [27]. We gathered the RSSI values in bins of 20λ travelling distance and calculated the average RSSI for each bin. All data processing and analyses were done based on the 100 byte packets.

Of interest for the characterization of the multilink shadowing effects were the absolute values of the simultaneous signal strengths, the joint distribution of the shadow fading process, and its autocorrelation function. In addition, the cross-correlation of the large scale fading process between different links was of interest. The large scale fading was estimated by subtracting the expected distance dependent channel gain from the measured (20λ) averaged channel gain. The maximum likelihood (ML) estimation with censored data samples [28,29] was used to estimate the channel gain parameters. All RSSI values of the communication links were compensated with respect to the TX output power and with the cable losses in the respective vehicles (see Table 1); the resulting values were defined as channel gains. The antennas and cables used for the trucks were seen as one unit; therefore, no cable loss was measured.

To study the packet success ratio (PSR), the received packets were first gathered in bin sizes of 1 s. Since the transceivers were transmitting packets with a 10 Hz rate, the number of received packets was divided by 10 to get the instantaneous PSR in each bin. Then, the corresponding average distance between the two vehicles for every 1 s bin was calculated. The average distance in this case was $d_{t}+d_{r}$, i.e., the sum of the two distances from the vehicles to the intersection center, also called “*Manhattan-distance*”. The PSR, as a function of the Manhattan-distance, was of primary interest for the analysis and to achieve this, a second binning was made, in which all average distances were collected in 5 m bin sizes. Then, the instantaneous PSR values were used to estimate an average PSR value for every 5 m bin.

Regarding censored samples, if there were lost packets in an NLOS communication link between the minimum, $d_{min}$, and maximum, $d_{max}$, of the Manhattan-distance, respectively, estimation of the distances for these censored samples was done by interpolation between successfully decoded packets. For large distances, beyond $d_{max}$, estimation of Manhattan-distances was done by the help of the positions of the successfully decoded packets by other TX vehicles in the group.

### 2.3. Path Loss Model

As mentioned in the introduction, an important use case for V2V communication in urban environments is the NLOS communication around corners in intersections, with the purpose of increased safety. This NLOS communication was modeled by Mangel et al. in [15], and the path loss model was there described by

(1) $$\begin{array}{c} PL\left(d_{t},d_{r},w_{r},x_{t},i_{s}\right)=3.75+i_{s}2.94+\left\{\begin{array}{cc} 10\log_{10}\left({\left(\frac{d_{t}^{0.957}}{{\left(x_{t}w_{r}\right)}^{0.81}}\frac{4\pi d_{r}}{\lambda }\right)}^{2.69}\right), & d_{r}\leq d_{b}\;\\ 10\log_{10}\left({\left(\frac{d_{t}^{0.957}}{{\left(x_{t}w_{r}\right)}^{0.81}}\frac{4\pi d_{r}^{2}}{\lambda d_{b}}\right)}^{2.69}\right), & d_{r}>d_{b}, \end{array}\right. \end{array}$$

where $d_{t}$, $d_{r}$, are the distances of TX and RX to intersection center, $w_{r}$ is the RX street width, and $x_{t}$ is the distance from TX to the facade. This model has, for a very long time, been the only or the most popular measurement-based model for path loss in street intersections. It is based on a large measurement effort but has its limitations, for example, it is not reciprocal. Two things contribute to this issue: (1) the exponents of $d_{t}$ and $d_{r}$ are different, and (2) if the intersection and vehicle locations are not symmetric, the values on $x_{t}$ and $w_{r}$ will be different when swapping the TX and RX vehicles. Another limitation is that the model does not consider whether the NLOS communication link between the two vehicles also is obstructed by other vehicles (OLOS). In addition, the TX-device used in [15] was not a car; instead, it was a tripod that was always fixed at a specific distance to the intersection center during the measurements. The values of exponents in the model would probably be different if the TX was also a car, since cars can affect the antenna pattern significantly (see Figure 2). Finally, censored values were not considered when estimating the parameters of the NLOS path loss model in [15].

**Figure 4:**
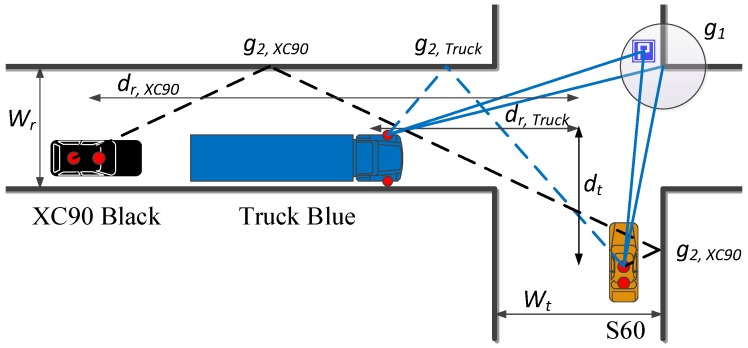
Description of the channel gain model for a typical non line-of-sight (NLOS) communication link between vehicles in an urban intersection. The solid blue lines represent single interactions from the center area of the intersection which are only “seen” by Truck Blue and the S60. The XC90 Black is blocked by Truck Blue and has OLOS towards the intersection center; therefore, it has no single interactions towards the S60. Dashed lines represent multiple interactions between the S60 and Truck Blue and between the S60 and XC90 Black, respectively.

To overcome the issues with reciprocity and the obstruction of communication links by other vehicles towards the intersection center, we propose the following channel gain model for NLOS communication at intersections:

(2) $$\begin{array}{c} G\left(d_{t},d_{r}\right){|}_{\mathrm{dB}}=\underset{\mathrm{Offset}}{\underset{︸}{10\log_{10}\left(m^{2}\right)}}+10\log_{10}\left(\underset{\mathrm{Single}\;\mathrm{interactions}}{\underset{︸}{{\left(g_{1}\frac{\lambda }{4\pi (d_{t}+d_{r})}\right)}^{2}}}+\underset{\mathrm{Multiple}\;\mathrm{interactions}}{\underset{︸}{{\left(g_{2}^{N}\frac{\lambda }{4\pi (d_{t}+d_{r})}\right)}^{2}}}\right)+\Psi_{\sigma }, \end{array}$$

where

$$\begin{array}{c} N=\max\{2\sqrt{\frac{d_{t}d_{r}}{w_{t}w_{r}}}-1,0\}, \end{array}$$

and where the widths of the streets at which the transmitting and receiving vehicles are located are denoted by $w_{t}$, $w_{r}$, and $d_{t}$, $d_{r}$ are the distances to the intersection center. The term $\Psi_{\sigma }$ represents the large scale fading and is modeled as a non-zero mean Gaussian process for each communication link and iteration (repetitive measurement runs in the same intersection). Further details are described in Section 3.4. The power contributions to the channel gain are modeled with two separate terms: the first term, which is denoted ‘single interactions’, models power contributions stemming from interactions near the intersection center. These interactions can typically be attributed to building corners or street signs located near the intersection center [19]. The second term, denoted ‘multiple interactions’, models the power contributions stemming from wall reflections. Here, *N* is the effective number of interactions. The equation is modified from that presented in [20], such that the number of interactions is non-integer and smoothly varying as a function of distance. We note that, in practice, there are, of course, often several single and higher order reflections present that contribute to the power. Therefore, $g_{1}$ and $g_{2}$ will actually describe a mean effective amplitude gain stemming from several components; $g_{1}$ is the mean effective amplitude gain of the contributions from the single interactions, whereas $g_{2}$ is the mean effective amplitude gain per interaction of the multiple interactions. The term *m* is a an offset for each link type, and is mostly attributed to differences in antenna height and differences in the combined TX and RX antenna patterns of each link pair. A schematic description of the channel gain model is shown in Figure 4.

## 3. Results

**Figure 5:**
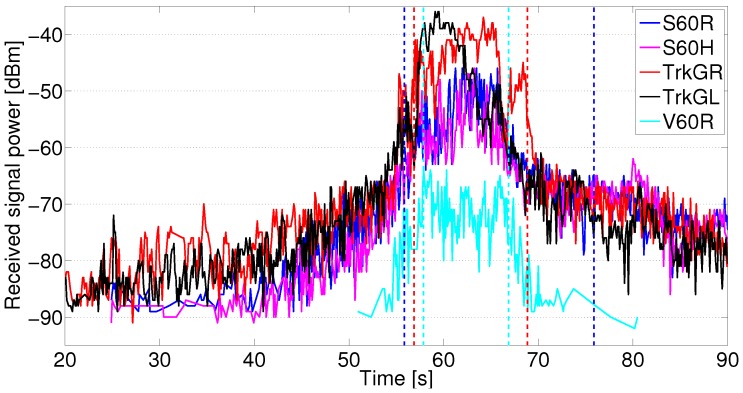
Received signal power at the XC90 Silver Roof antenna from all vehicles in group 2 during one of the measurement iterations. The dotted lines indicate when the XC90 Silver Roof antenna has line-of-sight (LOS) communication with S60 (blue) and Truck Gold (red). The cyan dotted lines indicate OLOS communication between the XC90 Silver Roof antenna and the V60 Roof antenna.

Using the antenna configurations at the six vehicles, 41 communication links were measured simultaneously. Out of these, the links between the vehicles in the different groups, in total, 25 communication links, were analyzed further. From all the measurements, nine iterations representing good repetitive iterations were used in the analysis, i.e., with no other vehicle(s) in the group and without large gaps between the vehicles in the groups. An example of received signal power vs. time in intersection Yngve is shown in Figure 5. Next, we present the analysis of the packet success ratio and path loss model estimation.

### 3.1. Packet Success Ratio

**Figure 6:**
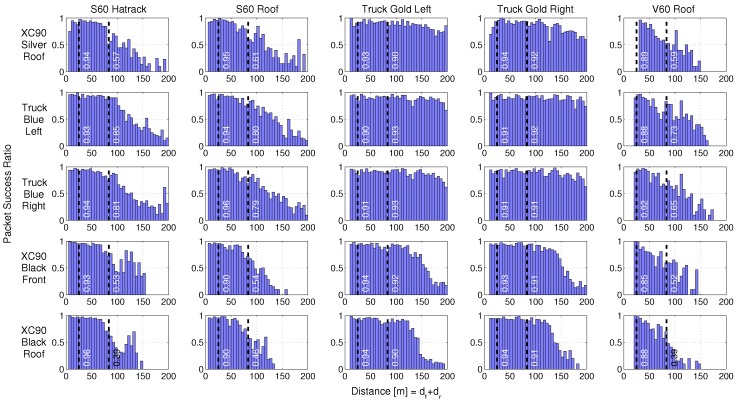
The packet success ratio vs. the Manhattan-distance ($d_{t}+d_{r}$) for all 25 analyzed simultaneous NLOS communication links between the two groups at intersection Yngve. The rows represent the vehicles in group 1, and the columns represent the vehicles in group 2. The first dotted line from the left is roughly the breaking distance (without reaction time) needed for two cars to avoid collision at a speed of 50 km/h (14 m/s). The second dotted line is the *"three-second safety distance recommendation"* by Swedish traffic schools to avoid a collision at the same speed, which corresponds to 83.3 m. The white numbers close to the dotted lines represent the packet success ratio of receiving a single packet transmitted within one second before the dotted lines, from each vehicle, respectively.

When designing the ITS-G5 protocol [30], large effort was made on the MAC-layer, to support as many as possible surrounding vehicles, without degradation of the PSR for each vehicle within the communication range. For example, a de-centralized congestion control (DCC) mechanism was implemented to keep the PSR high and channel access delay low, even in dense VANETs. However, the PSR does not only depend on the number of surrounding vehicles, it also depends on the distance between the two vehicles. When the energy of a received packet is below the sensitivity level of the receiver, the receiver cannot decode the packet and we have a lost packet. Figure 6 visualizes the PSR as a function of the Manhattan-distance of our measurements performed in intersection Yngve for all 25 communication links between the vehicles in the different groups.

As seen in Figure 6, the highest PSRs at long distances were observed for the truck-to-truck communication links and the Truck Gold to XC90 Silver link, which is the first car in group 1. The second best performances were the truck-to-car link and the communication between the two cars in the front of group 1 and group 2. The lowest PSR vs. distance values were observed for the last two cars in each group, which is natural since they were experiencing dual blocking through NLOS+OLOS communication most of the time. In one of the iterations, the S60 car was standing still very close to the intersection (<10 m), but was still under the NLOS condition. This resulted into a much longer communication range for the communication links between the S60 Hatrack and the two antennas on the XC90 Black, compared to the other iterations. That is why we saw an increased PSR at around 125 m of these two communication links.

One would expect PSR to be close to one at short distances. Possible explanations why this is not the case for any of the 25 communication links could be: (1) transceivers were transmitting packets at the same time; (2) destructive multipath self-interference, i.e., fading dips of at least 30–40 dB; (3) interference in the 5.9 GHz band from surrounding objects or from the vehicle itself; (4) the log equipment in the transceivers was not working properly; (5) something was wrong with the SW or HW of the transceivers; (6) the chipsets were not properly optimized for the vehicular environment experienced. The probability of alternatives 1–3 is low or very low. The alternatives 4–6 are more likely, but with no conclusive answer yet.

What do these PSRs mean in reality? Let us assume that two self-driving cars are driving towards the intersection Yngve at 50 km/h (14 m/s), and two human-driven cars are driving towards the same intersection at the same speed. The stop distance of a car is typically split into two parts [31]: the reaction distance and the break distance. The last part has many degrees of freedom (road friction, tires, break force, etc.) but, according to [32], the break distance on dry asphalt is 12.5 m for a car driving at 50 km/h. The reaction time is normally between 0.5–2 s for a driver, which gives a reaction distance between 7.0 m and 27.8 m. In total, the maximum stop distance is 40.3 m or 41.7 m according to the “*three-second safety distance recommendation*” by Swedish traffic schools to avoid collisions. In Figure 6, the two cases are marked by the dotted lines, and the numbers show the PSR of a single transmitted packet at the specific time instant to avoid a collision (more precisely, 0–1 s before the dotted lines). With a de-correlation distance of around 3.5 m the transceiver will, with a very rough approximation, have the possibility of independently receiving around four packets during one second, resulting in a total packet success ratio of $1-{\left(1-PSR\right)}^{4}$, e.g., the total PSR for the communication between XC90SR and S60H is 0.96. For basically all links, the vehicles will receive packets even before the critical distance and the overall packet success ratio will therefore be even higher. The specific numbers presented in Figure 6 should be used with care, but show interesting general behavior.

### 3.2. Channel Gain Model Estimation

To parameterize the channel gain model, estimation of the parameters $g_{1},g_{2},m,$ and σ of (2) was performed for five different NLOS link types, according to Table 2. The reason for this was that each link type has similar channel characteristics and behaviors. The truck-to-truck communication link had antenna heights of around 3 m. With these tall antenna heights, the communication link experienced fewer interactions from other surrounding cars. Almost the same was true for the truck-to-car communication link, where one side of the communication link experienced less interaction from the surrounding cars. The car-to-car link, where both TX and RX had LOS to the intersection center, experienced a strong contribution from the area of the intersection center. For the two last link types, at least one car was always obstructed by a truck. Therefore, these two link types, basically received no energy directly from the area of the intersection center, i.e., $g_{1}$ was very small. We note that the uncertainty (more specifically, the standard error) of the estimated parameters depends on the number of samples, the measurement sample distances, the amount of censored samples as well as the path loss model and its parameters [29]. In this paper, we did not derive the standard errors of the estimates for each link type, but leave that for future work.

**Figure 7:**
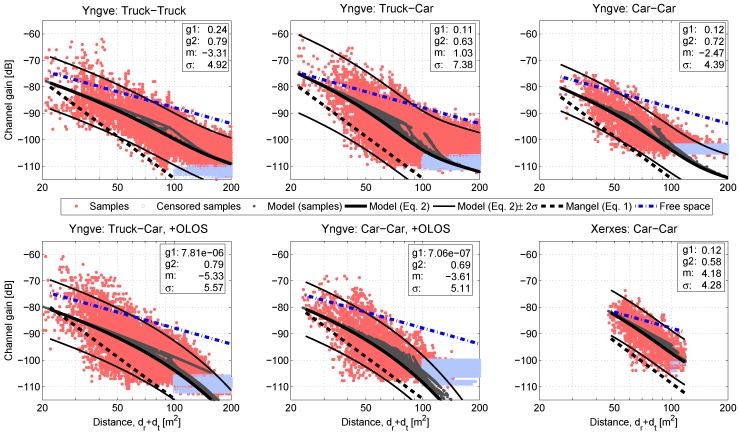
Channel gain vs. the Manhattan-distance ($d_{t}+d_{r}$) for all communication link types in intersection Yngve and the communication link type between the two front cars in intersection Xerxes (see Table 2. The estimated values, in decibels, of the parameters $g_{1},g_{2},m,$ and σ for the model (2) are shown in each subfigure. Both the Mangel-model (1) and the free space model are plotted for reference. The grey curves represent (2), based on the measured samples, and since our proposed model is based both on ($d_{t}+d_{r}$) and ($d_{t}\cdot d_{r}$), we actually have two dimensions of the distance influence. The lines showing the model (1), (2), and free space are based on fictive test scenarios where two vehicles are approaching Yngve or Xerxes and then stop. The fictive test scenarios use the estimated parameter values for each link type.

When estimating parameters of a path loss model, two important aspects need to be considered: (1) Is there a large range of sampled measurement distances? (2) Are there censored samples? Regarding the first aspect, the ratio between maximum and minimum distance of the intersection Yngve (taking the censored samples into account) was around 10, but not for Xerxes, as seen in Figure 7. As stated in [6], $d_{max}/d_{min}\geq 10$ is a good rule of thumb for the range of distances. Due to the limited maximum measurement distance in Xerxes, it was not possible to accurately estimate $g_{1}$. Instead, we assumed that this parameter was the same as in Yngve for the links with LOS towards the intersection and zero for the links with OLOS towards the intersection center. In addition, we studied the sensitivity of $g_{2}$ with regard to different values of $g_{1}$, and $g_{2}$ was not so sensitive.

Regarding the second aspect, as mentioned in Section 2.2, we considered censored samples if there were lost packets—the technique used in [28,29]. The channel gain value of the censored samples was equal to the lowest channel gain value of the sample size in that iteration +1 dB. The extra 1 dB was added since the sensitivity level was floating and had no sharp limit. Each measurement iteration had a different lowest level of channel gain; therefore, there is no clear censoring threshold in Figure 7. Censored samples with a Manhattan-distance below 100 m were seen as outliers caused by one of the six cases described in Section 3.1. Therefore, these outliers were not included when estimating the parameters of (2).

As seen in Figure 7, the channel gain of the measured samples for all link types and both intersections was lower than the free space model and generally higher than predicted by the Mangel model (1). For the vehicles with LOS to the intersection center, the contribution of the single interactions was on the same level as that of the multiple interactions. As an example, for the truck-to-car communication link in Yngve, the estimated parameters were $g_{1}$ = 0.11 and $g_{2}^{5}$ = 0.10. At Yngve, the parameter *N* had values in the range 0 to 12. With the strong single interactions from the center area of the intersection, the slope of the channel gain is reduced when the distance is increased, and we got some kind of S-shape for the channel gain with respect to distance. With the vehicles that had OLOS to the intersection center, the behavior was the opposite. There, the slope of the channel gain is increased when the distance is increased, forming an arc shape due to the small contribution from the single interactions. The values of all the estimated parameters of (2) are found in Table 3. When interpreting these results, one should keep in mind that, despite the fact that nine measurement iterations were performed and the intersections represent common structures of urban intersections, the parameters were influenced by the conditions in those specific intersections. The authors believe that the model structure is generally valid, but the specific parameter values will of course be affected by, e.g., street widths and building materials. Further validation in different intersections and cities around the world is therefore desirable, but outside the scope of this paper due to resource limitations.

### 3.3. Spatial Correlation of Shadow Fading

In urban environments, vehicles often shadow each other when the traffic is dense. The shadowing will influence the V2V communication, and the effect is called large scale fading. The shadowing process, or the large scale variation, in (2) can be well described by a Gaussian random variable, $\Psi_{\sigma }$ with a standard deviation σ and a non-zero random mean for each iteration. Once a vehicle is shadowed, it will be so for some traveled distance interval. During this distance interval, one could say that if one packet fails, there is a higher probability that the next packet will fail as well. The correlation distance, $d_{c}$, roughly describes for how long the distance interval of the large scale fading process can be assumed to be static. The large scale fading, $\Psi_{\sigma }$, is achieved by subtracting the distance dependent mean from the overall channel gain. Then, the spatial autocorrelation of the large scale fading can be written as,

(3) $$\begin{array}{c} r\left(\Delta d_{i}\right)=E\left\{\Psi_{\sigma }\left(d_{i}\right)\Psi_{\sigma }\left(d_{i}+\Delta d_{i}\right)\right\}, \end{array}$$

where $d_{i}$ is the Manhattan-distance between TX and RX. Since the traffic was dense during the measurements, the speeds of the test vehicles varied significantly, resulting in the received power levels being irregularly sampled in distance. This needed to be considered when estimating the spatial sample autocorrelation of the large scale fading. Therefore, the traveled distance, $d_{i}$, was collected in Δd bins, and the method as in [6] was used.

The autocorrelation of the shadowing process can be approximated by a well-known model proposed by Gudmundson [33], based on a negative exponential function:

(4) $$\;r\left(\Delta d_{i}\right)=\sigma^{2}e^{-\left|\Delta d_{i}\right|/d_{c}}=\sigma^{2}\rho \left(\Delta d_{i}\right).$$

In the Gudmundson model, $d_{c}$ is defined as the value of $\Delta d_{i}$ at which the value of the autocorrelation function $\rho (\Delta d_{i})$ has decreased to 1/*e*.

**Figure 8:**
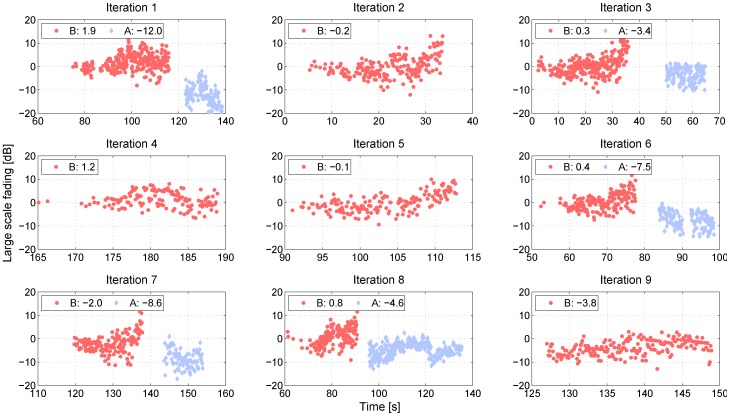
Large scale fading (LSF) of the NLOS communication link between XC90 Black Front and Truck Gold Right at intersection Yngve before (A) and after (B) the intersection. In all of the iterations, there was NLOS communication before the intersection and in some of the iterations, there was also NLOS communication after the intersection, i.e., when the XC90 Black car had passed the intersection. The numbers in each subfigure represent the offset between the mean of the specific LSF process from zero.

**Figure 9:**
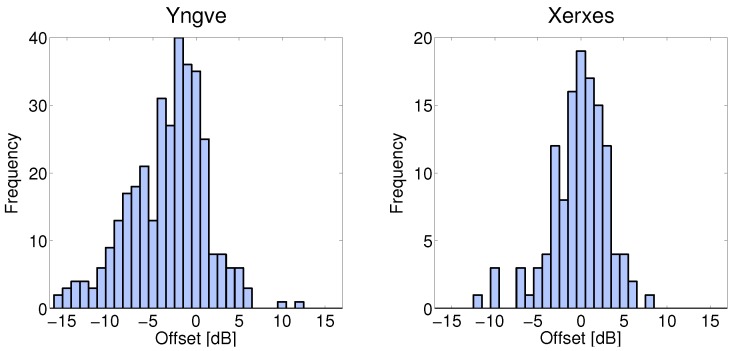
To the left is a histogram of the iteration offset of the large scale fading ($\Psi_{\sigma }$), both before and after intersection Yngve. To the right is the same offset of intersection Xerxes, but with only measurements before the intersection, since no measurements were performed after the intersection.

In Figure 8, the large scale fading for the NLOS communication link between XC90 Black Front antenna and the Truck Gold Right antenna is presented for the nine iterations at intersection Yngve. As seen in the figure, the large scale fading process is not an zero-mean process, instead there is an offset between the iterations, as well within some of the iterations. For iterations, 1, 3, 6, 7, and 8, the XC90 Black car was passing by the intersection. The large scale fading process before the intersection is marked by, B, and the large scale fading process after the intersection is marked by, A. (In the other iterations, the XC90 Black car was also passing by the intersection, but in these iterations, we only had NLOS communication before the intersection). Before the intersection, the offsets of the large scale fading were between −3.8 dB to 1.9 dB, and afterwards, the offsets were between −12.0 dB and 3.4 dB. The offsets can be explained by the traffic situation during the specific iteration and the antenna gain pattern of the two involved vehicles. In particular, the antenna gain pattern can explain the difference in offset within an iteration. As seen in Figure 2, the antenna gain varied between −5 dBi and 0 dBi in the sector from 0 to 270 degrees. After having passed the intersection, the antenna gain varied between −15 dBi and −5 dBi; therefore, we saw a negative shift in the shadowing process before and after the intersection. The offset values in Figure 8 are the differences between the specific large scale fading process for that iteration, even within an iteration, and the path loss model (2), which was estimated for all the communication links belonging to the link type Truck-to-Obstructed car. The offsets of the large scale fading, $\Psi_{\sigma }$, for all NLOS links for both of the intersections are presented as two histograms in Figure 9. The offset can also be modeled as a Gaussian distribution, with μ = −3.2 dB, σ = 4.5 dB for Yngve, and μ = −0.2 dB, σ = 3.3 dB for Xerxes. The reason for the negative shift of the histogram in Yngve is the number of censored samples.

**Figure 10:**
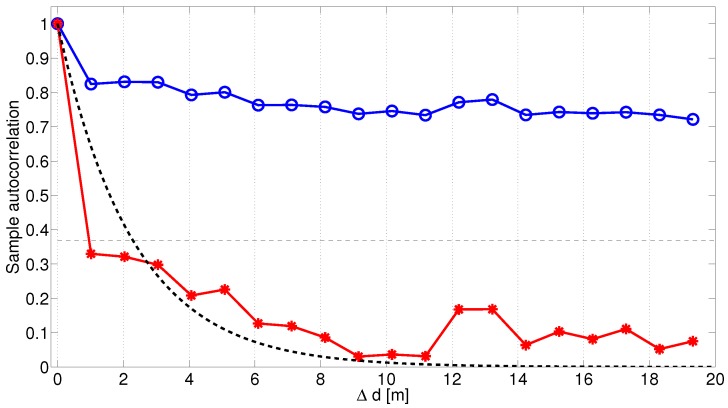
Auto-correlation function for the large scale fading, for both measurements and the model according to (4) of the communication link between XC90 Black Front and Truck Gold Right at intersection Yngve. Two measurement curves are seen in the figure: (1) where the large scale fading processes in this link are non-zero-mean process (blue with circle). (2) where the offset values have been subtracted from the large scale fading processes, i.e., a zero-mean process is achieved (red with stars).

**Figure 11:**
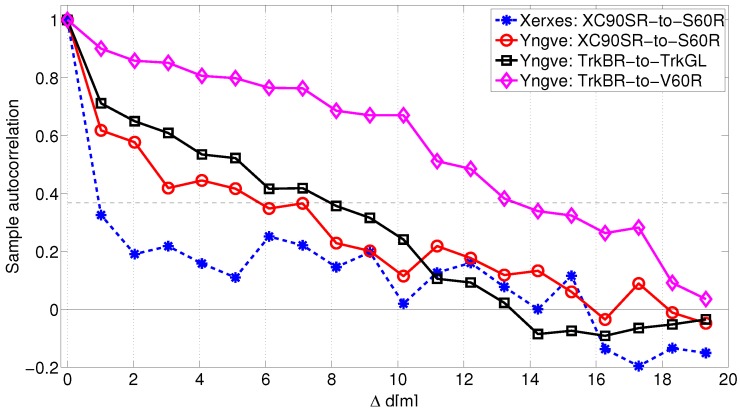
Auto-correlation as a function of traveled distance for four out of the 25 communication links.

**Figure 12:**
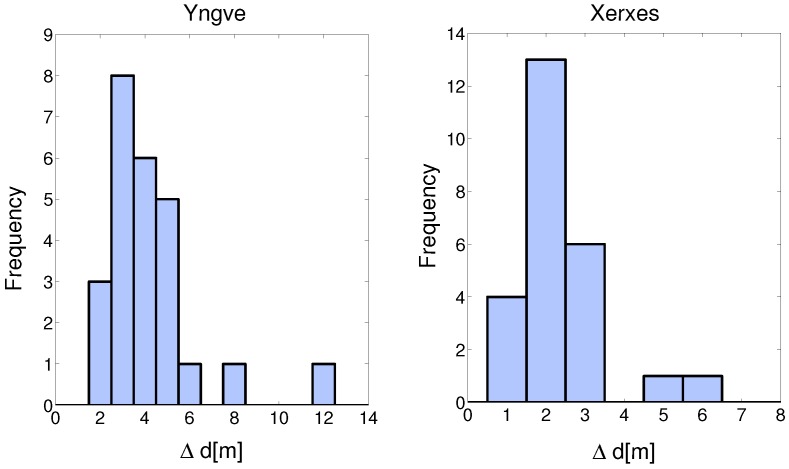
Histograms of the de-correlation distances of all NLOS communication links in the intersections Yngve and Xerxes.

An offset of the link gain affects the sample auto-correlation significantly, as visualized in Figure 10. The blue curve with the circles is the estimated auto-correlation function where the large scale fading process is not a non-zero-mean process, i.e., the offset values in Figure 8 are not subtracted. The red curve with the stars is when the subtraction is performed and the shadowing process is a zero-mean process. The black dotted line is the modeled autocorrelation of the zero-mean large scale fading process. For the link between XC90 Black Front antenna and Truck Gold Right antenna, the de-correlation distance was 2.3 m. For (4) we focused our attention on modeling the initial decay and therefore, values of 0≤Δd≤5 m were used as input to the ordinary least square estimator of (4). In Figure 11, the auto-correlation of a few other of the 25 communication links are shown. The histograms of the de-correlation distance of intersections Yngve and Xerxes are shown in Figure 12, where the median de-correlation distance in intersection Yngve is 3.7 m and in Xerxes is 2.2 m. The de-correlation distances are within the same range as in [16,34].

### 3.4. Multilink Shadowing Correlation

In the literature, the shadow fading cross-correlation has often been modeled as function of only space [35,36], even though the multilink shadowing is a complex mechanism both in space and time. In [6], the cross-correlation between two links had a common node (the TX) as a function of the distance between the two receiver vehicles was analyzed. Here, we had to restrict the analysis to more or less fixed inter vehicle distances within the groups. In any case, the analysis of the cross-correlation of the multilink shadowing mechanism is of value as there might be some non-negligible cross-correlation of the shadowing process when two RX vehicles receive the same message from one TX vehicle or vice versa in the VANET.

The cross-correlation can be written as

(5) $$\begin{array}{c} \rho_{L1,L2}=E\left\{\left(L1_{t}-\mu_{L1}\right)\left(L2_{t+\tau }-\mu_{L2}\right)\right\} \end{array}$$

where the large scale fading values of link 1 and link 2 are represented by L1 and L2, respectively.

**Figure 13:**
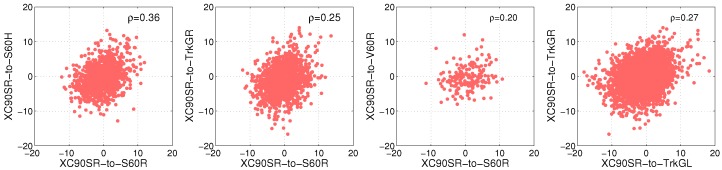
The multilink shadowing correlation between different links at intersection Yngve. The x-axis for the first three subfigures represents the link between the XC90 Silver Roof to the S60 Roof; the y-axis represent the communication between the XC90 Silver roof and one antenna on each vehicle in group 2. The last subfigure, show the cross-correlation of the links between the XC90 Silver Roof and the two antennas on Truck Gold.

The estimated cross-correlation, $\rho_{L1,L2}$, between four different communication link pairs is presented in Figure 13 for intersection Yngve. As seen in the scatter plots, the cross-correlation between the large scale fading processes of different links was rather small. What is very interesting to note is that the rightmost and leftmost subfigures show the cross-correlation of the large scale fading between two links from the same vehicle but for two different antennas. If the antenna patterns were very similar, e.g., with dipole antennas located 2 m above the vehicle’s roof, one would expect a cross-correlation of close to one. However, since the antenna gain pattern differed significantly between all mounted antennas on the vehicles, the cross-correlation became marginal, which also was the case in [6]. The cross-correlation between links involving different vehicles, as shown in the two subfigures in the middle of Figure 13, was also small. Due to these low values of cross-correlation with respect to the multilink shadowing, this complex mechanism can be neglected when performing simulations of VANETs, given that a proper channel gain model, such as (2), is used.

## 4. Discussion

It is quite remarkable that so little attention has been paid to the development of NLOS path loss models for V2V communication in urban intersections, since this scenario is one of the most critical safety scenarios for V2V safety applications. The Mangel model [15] is an exception, providing an NLOS model based on extensive measurements, and is by far the most used one today. However, there are limitations with the Mangel model with respect to reciprocity and because the model does not consider other vehicles obstructing the NLOS communication link. These two limitations were considered in the analysis in this paper of V2V measurements between six vehicles in the city of Gothenburg, Sweden. We proposed a measurement-based reciprocal V2V NLOS path loss model (2), for intersections, comprising single interactions from the intersection center area together with multiple interactions from the building walls. When the communication link was obstructed by other vehicles, the contribution from the single interactions was insignificant. On the other hand, when both the communicating vehicles had LOS towards the intersection, it was the single interaction factor that dominated at large distances. The channel gains in our measurements were generally higher compared to what was predicted with the Mangel model. With our proposed model, the communication links will have longer ranges compared to the cases using the Mangel model; another aspect is that interference levels from other vehicles will increase.

We also presented measured packet success ratios, PSRs, for 25 simultaneous communication links in the intersections. The results show that the probability of receiving information from another vehicle around the corner approaching the same intersection is rather high before the moment when it is too late to take action to avoid a collision. By using V2V communication as a complement to the on-board sensors (e.g., radars, cameras, and lidars), traffic safety can be increased in intersections, even during dense traffic when vehicles are shadowing each other.

In urban environments, dense traffic is common, resulting in vehicles shadowing each other, and by that, influencing the V2V communication performance. The de-correlation distance is a measure of how long the shadowing process can be assumed to be static. Our measurements highlight that the autocorrelation as a function of traveled distance has very different behavior depending how well the path loss model represents the measured samples. By using our proposed channel gain model (2), the de-correlation distance was around 2–4 m. In our model, the large scale fading for a single measurement run (iteration), represented by the random variable $\Psi_{\sigma }$, is not a zero-mean Gaussian process, which is a common assumption. Instead, $\Psi_{\sigma }$, is non-zero mean Gaussian process. The mean of $\Psi_{\sigma }$ has a Gaussian distribution that represents the differences in the particular traffic situation and the gain of the involved antennas for the particular communication link. By using the proposed channel gain model (2), the analysis showed that the cross-correlation between different links was small, even for communication links with antennas located at the same vehicle.

## 5. Conclusions

The overall conclusion of our analysis of path loss, de-correlation distance, and multilink shadowing effects of V2V communication in an urban environment, is that geometry-based models should be used for VANET simulations. By doing so, and using an appropriate channel gain model with some randomness of the parameter settings to reflect different vehicle types, the de-correlation distance can be close to what is experienced in reality and cross-correlation can be neglected. The latter will make the implementation of realistic models in VANET simulators much easier.

We are aware that our channel gain model is only based on measurements in two intersections of different kinds, though the measurement campaign included six vehicles and nine iterations. To make our proposed model generic, the model needs to be further validated, which we leave as future work.

## Figures and Tables

**Table 1:** Measurement Parameters.

Parameter	Value(s)
Standard	IEEE 802.11p
Center frequency	5.9 GHz
Data rate	6 Mbit/s
Packet rate	10 Hz
Packet size	100, 500, and 1500 bytes every 100 ms.
Congestion control	Decentralized congestion control not implemented.
TX output power	+23 dBm, limit in [26], +23 dBm/MHz e.i.r.p
RX sensitivity	−97 dBm @ Packet Error Rate of 10 %
Polarization	Vertical
S60 Roof ant. (S60R)	Height 144 cm, cable loss, 2.5 dB.
S60 Hatrack ant. (S60H)	Height 110 cm, cable loss, 4.7 dB.
V60 Roof ant. (V60R)	Height 144 cm, cable loss, 5.0 dB.
XC90 Silver Roof ant. (XC90SR)	Height 169 cm. cable loss, 4.6 dB.
XC90 Black Roof ant. (XC90BR)	Height 169 cm. cable loss, 4.2 dB.
XC90 Black Front ant. (XC90BF)	Height 146 cm. cable loss, 1.25 dB.
Truck Blue Left ant. (TrkBL)	Height 301 cm. cable loss, NA.
Truck Blue Right ant. TrkBR)	Height 302 cm. cable loss, NA.
Truck Gold Left ant. (TrkGL)	Height 304 cm. cable loss, NA.
Truck Gold Right ant. (TrkGR)	Height 301 cm. cable loss, NA.

**Table 2:** Definition of NLOS link types.

Link Type	Communication Link
Truck-to-Truck	Truck Blue Left ant. to Truck Gold Left ant.
	Truck Blue Left ant. to Truck Gold Right ant.
	Truck Blue Right ant. to Truck Gold Left ant.
	Truck Blue Right ant. to Truck Gold Right ant.
Truck-to-Car	Truck Blue Left ant. to S60 Hatrack ant.
	Truck Blue Left ant. to S60 Roof ant.
	Truck Blue Right ant. to S60 Hatrack ant.
	Truck Blue Right ant. to S60 Roof ant.
	XC90 Silver Roof ant. to Truck Gold Left ant.
	XC90 Silver Roof ant. to Truck Gold Right ant.
Car-to-Car	XC90 Silver Roof ant. to S60 Hatrack ant.
	XC90 Silver Roof ant. to S60 Roof ant.
Truck-to-Obstructed car	Truck Blue Left ant. to V60 Roof ant.
	Truck Blue Right ant. to V60 Roof ant.
	XC90 Black Front ant. to Truck Gold Left ant.
	XC90 Black Front ant. to Truck Gold Right ant.
	XC90 Black Roof ant. to Truck Gold Left ant.
	XC90 Black Roof ant. to Truck Gold Right ant.
Car-to-Obstructed car	XC90 Black Front ant. to S60 Hatrack ant.
	XC90 Black Front ant. to S60 Roof ant.
	XC90 Black Front ant. to V60 Roof ant.
	XC90 Black Roof ant. to S60 Hatrack ant.
	XC90 Black Roof ant. to S60 Roof ant.
	XC90 Black Roof ant. to V60 Roof ant.
	XC90 Silver Roof ant. to V60 Roof ant.

**Table 3:** Estimated parameters for the proposed path loss model (2) in the two intersections.

Link Type	Yngve				Xerxes			
	$g_{1}$	$g_{2}$	*m*	σ	$g_{1}$	$g_{2}$	*m*	σ
			[dB]	[dB]			[dB]	[dB]
Truck-to-truck	0.24	0.79	−3.31	4.92	0.24	0.67	1.23	4.58
Truck-to-car	0.11	0.63	1.03	7.38	0.11	0.53	4.60	5.26
Car-to-car	0.12	0.72	−2.47	4.39	0.12	0.58	4.18	4.28
Truck-to-Obstructed car	$7.81\times 10^{-6}$	0.79	−5.33	5.57	0	0.60	1.72	4.35
Car-to-Obstructed car	$7.06\times 10^{-7}$	0.69	−3.61	5.11	0	0.49	5.86	4.75
